# Mother’s Curse effects on lifespan and aging

**DOI:** 10.3389/fragi.2024.1361396

**Published:** 2024-03-08

**Authors:** Suzanne Edmands

**Affiliations:** Department of Biological Sciences, University of Southern California, Los Angeles, CA, United States

**Keywords:** aging, longevity, mitochondria, mitonuclear, Mother’s Curse, senescence, sex differences, sex-specific selective sieve

## Abstract

The Mother’s Curse hypothesis posits that mothers curse their sons with harmful mitochondria, because maternal mitochondrial inheritance makes selection blind to mitochondrial mutations that harm only males. As a result, mitochondrial function may be evolutionarily optimized for females. This is an attractive explanation for ubiquitous sex differences in lifespan and aging, given the prevalence of maternal mitochondrial inheritance and the established relationship between mitochondria and aging. This review outlines patterns expected under the hypothesis, and traits most likely to be affected, chiefly those that are sexually dimorphic and energy intensive. A survey of the literature shows that evidence for Mother’s Curse is limited to a few taxonomic groups, with the strongest support coming from experimental crosses in *Drosophila*. Much of the evidence comes from studies of fertility, which is expected to be particularly vulnerable to male-harming mitochondrial mutations, but studies of lifespan and aging also show evidence of Mother’s Curse effects. Despite some very compelling studies supporting the hypothesis, the evidence is quite patchy overall, with contradictory results even found for the same traits in the same taxa. Reasons for this scarcity of evidence are discussed, including nuclear compensation, factors opposing male-specific mutation load, effects of interspecific hybridization, context dependency and demographic effects. Mother’s Curse effects may indeed contribute to sex differences, but the complexity of other contributing factors make Mother’s Curse a poor general predictor of sex-specific lifespan and aging.

## Introduction

Sex differences in lifespan and rates of aging are widespread, and the evolutionary basis of these differences are poorly understood (Marais et al., 2018; Bronikowski et al., 2022; Marais and Lemaître, 2022). At least three, non-mutually exclusive explanations have been advanced. One explanation is that the sex with unguarded sex chromosomes dies younger. That is, both XY males and ZW females bear a reduced sex chromosome, allowing deleterious recessive mutations on the larger chromosome to go unmasked. There is support for this hypothesis across a broad range of taxa (Xirocostas et al., 2020), but it cannot explain sexually dimorphic lifespan in taxa lacking sex chromosomes. A second explanation involves sexual selection (Adler and Bonduriansky, 2014). That is, the sex with greater competition for mates (usually males) is expected to favor a “live fast, die young” strategy that sacrifices longevity for reproduction. While there is certainly support for such tradeoffs (Hunt et al., 2004; Adler and Bonduriansky, 2014), there are also major exceptions (Keller and Genoud, 1997; Markow, 2011). A third explanation is the sex-specific selective sieve, also known as the “Mother’s Curse” hypothesis (Frank and Hurst, 1996; Gemmell et al., 2004). According to this hypothesis, mothers may curse their sons with deleterious mitochondria, because maternal inheritance makes selection blind to mitochondrial mutations that harm only males.

The Mother’s Curse (MC) hypothesis is a potential explanation for male-specific aging in all taxa with maternal inheritance of mitochondria, which includes the vast majority of eukaryotes. Mitochondrial DNA (mtDNA) is particularly prone to accumulating deleterious mutations due to uniparental inheritance, haploidy, lack of sexual recombination and low effective population size (Hoekstra, 2000; Neiman and Taylor, 2009). Further, because mitochondrial genomes are present in many copies per cell, within-individual selection can promote selfish mitochondrial haplotypes that are good at proliferation but bad for the host (Havird et al., 2019). And these deleterious and selfish haplotypes will be unopposed by selection if their deleterious effects are limited to males.

**FIGURE 1:**
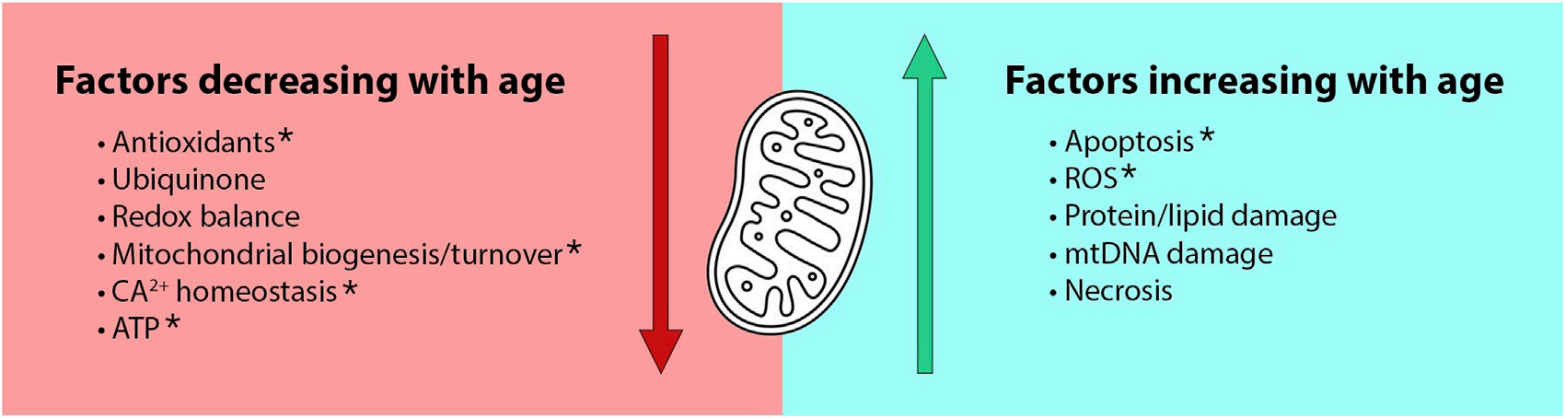
The Mother’s Curse hypothesis is an attractive explanation for male-specific aging due to known age- and sex-biased functions in mitochondria. Age-dependent mitochondrial defects include both decreased benefits and increased detriments in older organisms (Wang and Hekimi, 2015; Mendoza and Karch, 2022). Many of these functions are also sex-dependent. For example, functions denoted by asterisks are known to be aggravated by declines in estrogen (Ventura-Clapier et al., 2017).

The conserved function of mitochondria and their established role in aging makes MC a particularly attractive explanation for female-biased longevity. The modern mitochondrial theory of aging descends from the free radical theory of aging (Harman, 1956) which attributes aging to the gradual accumulation of oxidative cellular damage. Over time, this theory progressed to focus on the role of mitochondria in a positive feedback loop in which mitochondria produce free radicals that damage mtDNA, leading to mitochondrial dysfunction and accelerated production of reactive oxygen species (ROS) (Sanz and Stefanatos, 2008). Recent work suggests a more complex relationship between mtROS and aging, due to growing evidence for the beneficial regulatory effects of ROS and for a hormetic relationship between ROS and lifespan (Finkel and Holbrook, 2000). Many now view mitochondrial dysfunction as just one of several hallmarks of aging (López-Otín et al., 2013), with many mitochondrial defects being correlated with age (Wang and Hekimi, 2015; Mendoza and Karch, 2022). These defects include age-related increases in mtROS, apoptosis, necrosis and damage to mtDNA lipids and proteins, as well as age-related decreases in ATP production, redox balance, ubiquinone, calcium homeostasis, and mitochondrial biogenesis and turnover (Figure 1).

If MC effects are widespread, this may allow insights into conserved mechanisms of sex-specific lifespan and aging. Here I review patterns to be expected under the hypothesis, traits most likely to be affected, and potential medical implications. I also review evidence for and against the MC hypothesis, as well as factors that may limit or even reverse the curse.

## Patterns expected under Mother’s Curse

### Expect longer lifespan in females

Mother’s Curse has been invoked to explain the common pattern of females outliving males (Frank and Hurst, 1996; Camus et al., 2012). It is well established that human females live longer than males across timepoints and geographic locations, and this female advantage extends to the majority of mammals (Austad and Fischer, 2016; Xirocostas et al., 2020). However, short male lifespan may also be driven by the expression of deleterious recessive alleles in hemizygous XY males. Indeed, males tend to have longer lifespans in birds and butterflies, where females are the hemizygous (ZW) sex (Austad and Fischer, 2016; Xirocostas et al., 2020). The potential role of MC in reducing male lifespan may also be confounded with sexual selection, where males may sacrifice longevity for reproductive opportunities (Adler and Bonduriansky, 2014).

### Expect higher mitochondrial variance for trait expression in males

Under maternal inheritance, the effect of mutations on males is immune to selection, allowing mutations with the full spectrum of male-specific effects to pass through the selective sieve. As a consequence, variation in mitochondrial haplotypes is predicted to cause greater variation in trait expression in males than females (Dowling and Adrian, 2019). This prediction can be tested by placing different mitochondrial haplotypes onto the same nuclear background by breeding, and measuring the mitochondrial coefficient of variance for traits of interest in each sex. Such an approach is particularly effective for documenting the “weak form” of MC (Dowling and Adrian, 2019; Havird et al., 2019) which is the original formulation of the hypothesis by Frank and Hurst (1996) wherein male-harming mtDNA mutations are neutral or nearly neutral in females. Importantly, higher trait variation in XY males can also be caused by exposure of recessive alleles on the X chromosome, rather than mitochondrial effects.

### Expect negative intersexual genetic correlations

A third expectation under Mother’s Curse is a negative intersexual genetic correlation, such that mitotypes have opposite fitness consequences in the two sexes. This expectation is specific to the “strong form” of MC (Dowling and Adrian, 2019) in which male-harming mitochondrial mutations are beneficial to females. The subset of sexually antagonistic mutations that are beneficial in females can be expected to be rapidly promoted by natural selection (Unckless and Herren, 2009). The existence of intersexual genetic correlations can be tested by creating a panel of different mitotypes on the same nuclear background (as above), measuring traits of interest, and testing for intersexual correlations among mitotypes. While the evolution of sexual antagonism may be inevitable in species with separate sexes (Connallon and Clark, 2014), the evidence for sexually antagonistic mitochondrial mutations is somewhat limited. Such mutations have long been recognized in plants, where mitochondrial mutations in hermaphrodites have been found to increase female fertility while rendering males sterile, a phenomenon known as cytoplasmic male sterility (Lewis, 1941; Budar et al., 2003). In animals, recent studies have also found mitotypes with sexually antagonistic effects on both fertility and viability (Dowling and Adrian, 2019). As with the previous two patterns, negative intersexual correlations can also be caused by sex chromosome effects, rather than mitochondrial effects. Indeed, work on *Drosophila* has shown sexually antagonistic effects that are consistent with Mother’s Curse but difficult to disentangle from X chromosome effects (Rand et al., 2001). Given that X chromosomes are vastly larger than mitochondrial genomes, sex chromosome effects could be large.

### Expect mitonuclear mismatch to cause greater fitness problems in males

Mitochondrial function relies on tight coordination between gene products encoded by both the mitochondrial and nuclear genomes (Bar-Yaacov et al., 2012). This coordination is critical for ATP production, as well as for replication, transcription and translation of mtDNA. The existence of such inter-genome coevolution is supported by numerous studies in which mismatched mitonuclear hybrids suffer reduced fitness (Burton and Barreto, 2012; Estes et al., 2023). While disruption of coevolved mitonuclear complexes can be expected to impact both sexes, mismatched males may incur the additional loss of nuclear mutations that compensate for male-specific mitochondrial effects. This prediction can be tested by measuring sex-specific fitness in matched vs mismatched mitonuclear hybrids, particularly in maternal vs paternal backcross hybrids (Ellison and Burton, 2008) which have different mitotypes on an equivalent nuclear background, assuming minimal selection.

## Traits expected to be affected by Mother’s Curse

### Strongest effects for sexually dimorphic traits under high energy demand

Male-harming mitochondrial mutations are expected to accumulate more rapidly for the most sexually dimorphic traits. Mitochondrial mutational load has therefore been predicted to have greater effects on fertility than viability, since viability genes are thought to be largely shared between the sexes (Hollocher and Wu, 1996; Turelli and Orr, 2000), allowing males to profit from selection on females. Males are expected to lose the benefit of female-specific selection as the level of sexual dimorphism increases or the level of intersexual genetic correlation decreases. Because mitochondria provide most of the energy used by eukaryotic cells, traits and tissues with high energy demands are expected to be particularly vulnerable to mitochondrial limitations. In line with this prediction, mitochondrial dysfunction is particularly problematic for reproduction and for metabolic and degenerative diseases, with strong manifestations in energy-reliant tissues such as the brain, eye, muscle, heart and endocrine system (Jansen and Burton, 2004; Wallace et al., 2010; Chen et al., 2023). Mitochondrial dysfunction may be further aggravated by metabolically-demanding conditions, such as high temperature (Lane, 2011) or developmental stages with high growth rates (Hoekstra et al., 2018).

Mother’s Curse effects are therefore expected to be strongest for traits that are highly sexually dimorphic and also under high energy demand. Sperm traits fit both these criteria, and indeed have been a major focus of Mother’s Curse investigations. While mature oocytes may contain over 150,000 mtDNA copies, sperm often contain only −100 (Wai et al., 2010). This, coupled with sperm’s high energetic requirements, means that male fertility may be compromised if even a fraction of the mtDNA bears harmful mutations (Gemmell et al., 2004; Smith et al., 2010). In one of the first tests of the MC hypothesis, mitotypes were found to associate with sperm motility in human males (Ruiz-Pesini et al., 2000). However subsequent studies have failed to find mtDNA effects on human sperm motility (Pereira et al., 2007; Mossman et al., 2012) and the link between mtDNA and sperm quality appears to be complex (Boguenet et al., 2021). Even if mitotype effects on sperm function can be convincingly shown, it has been argued (Mossman et al., 2016a) that such sex-limited traits provide a poor test of the MC hypothesis, since they cannot be tested in females. More rigorous tests of the hypothesis would involve traits expressed in both sexes.

### Expectations for lifespan and aging

While MC effects may be more prominent in the most sexually dimorphic traits like reproduction, they may also manifest in traits associated with lifespan and senescence. Indeed, many age-related changes in mitochondrial function (Figure 1) also show sex differences, particularly for traits associated with metabolism. For mitochondria in most human tissues, estrogen has been found to increase mitochondrial biogenesis, antioxidant defenses, ATP production, oxidative capacity, fatty acid utilization and calcium retention capacity, and to decrease the release of ROS and pro-apoptotic factors (Ventura-Clapier et al., 2017). Mitochondria in human females have also been reported to show greater reliance on lipids over proteins as a fuel source (Demarest and McCarthy, 2015; Miotto et al., 2018), lower mitophagy under nutritional stress (Demarest and McCarthy, 2015), caspase-dependent apoptosis (Demarest and McCarthy, 2015), and lower sirtuin maintenance with aging (Barcena De Arellano et al., 2019). Given the comprehensive sex differences in age-related mitochondrial function, males would be at greater risk for a wide range of aging pathologies if mitochondria are optimized for females.

## Medical implications

### Sex-specific medicine

MtDNA mutations underlying disease were first described in 1988, and several hundred more pathogenic mtDNA mutations have been reported subsequently (Greaves et al., 2012). Many of these mitochondrial pathologies involve a chronic state of insufficient energy. The best known male-biased disease linked to mtDNA is Leber’s hereditary optic neuropathy (LHON), which affects approximately three times as many men as women (Poincenot et al., 2020). MtDNA variants have also been implicated in the expression of sex-biased, late-onset diseases such as schizophrenia and Parkinson’s (Hudson et al., 2014). Many other mitochondrial diseases may well be sex-biased, but have not been fully documented, due in part to the complications of strong tissue-specific mitochondrial function (Ventura-Clapier et al., 2017) and incomplete penetrance caused by heteroplasmy (Greaves et al., 2012), wherein cells contain both pathogenic and wildtype mtDNA. The policy of addressing sex as a biological variable, instituted by the National Institutes of Health (NIH) in the 1990s (Clayton, 2016), may help document the frequency of sex-biased disease, including those involving male-harming mitochondrial mutations, and lead to more effective therapies.

### Mitochondrial replacement therapy

The transmission of a mitochondrial disease from a woman to her child could be prevented by mitochondrial replacement therapy (MRT), a method of *in vitro* fertilization in which faulty mtDNA is replaced by healthy mtDNA from a donor female, effectively creating a three-parent embryo (Wolf et al., 2015). Among the risks of this method is the potential for mitonuclear mismatch between the mtDNA from the donor parent and the nuclear DNA from the other two parents. Human mitochondrial donation became legal in the United Kingdom in 2015. In 2014, the U.S. Food and Drug Administration convened an Advisory Committee to discuss the MRT issue, and advocated a cautious approach with extensive safeguards and initial restriction to male embryos, so that the donor DNA would not be passed on to the next generation (Claiborne et al., 2016; Claibourne et al., 2016). If MC mutations are common in humans, this “cautious” approach of restriction to male embryos may be particularly problematic, since males will suffer greater harm from disrupted mitonuclear interactions. At present, the United Kingdom and Australia are the only countries that expressly authorize mitochondrial replacement for reproduction (Noohi et al., 2022).

## Evidence

### Taxonomic patterns

Although the ubiquity of maternal mitochondrial inheritance should make most eukaryotes vulnerable to the Mother’s Curse, evidence for the hypothesis is limited to relatively few taxa (Table 1), with many of these same taxa also showing contradictory results (Table 2). Male-harming mitochondrial mutations have certainly been documented in flowering plants, where mtDNA mutations have been found to block pollen production and thereby turn hermaphrodites into females, a phenomenon known as cytoplasmic male sterility which was reported as early as Lewis, 1941. Historically it was predicted that male-harming mutations such as those found in plants would not persist in the highly streamlined mitochondrial genomes found in most animals (Camus and Dowling, 2018). When such cases were ultimately found in animals the phenomenon was later christened “Mother’s Curse” (Gemmell et al., 2004). In animals, support for the hypothesis (Table 1) has been reported in *Drosophila*, seed beetles, copepods, birds, hares, mice and humans. Notably, all but one of the species in Table 1 have sex chromosomes (the exception is the copepod *Tigriopus californicus*), which can create effects that are difficult to distinguish from MC effects. The bulk of the evidence for Mother’s Curse comes from work on *Drosophila*, where support has been found for the three strongest tests of the hypothesis: higher mitochondrial trait variance in males (Innocenti et al., 2011; Camus et al., 2012; Wolff et al., 2016; Aw et al., 2017; Nagarajan-Radha et al., 2020; Carnegie et al., 2021), negative intersexual correlations (Camus and Dowling, 2018; Nagarajan-Radha et al., 2020), and more deleterious effects of mitonuclear mismatch in males (Sackton et al., 2003). However, broad taxonomic surveys, albeit with limited power, have failed to find support for the hypothesis (Table 2). This includes a study of 108 mammal species (Cayuela et al., 2023) which found no sex differences in the influence of mtDNA mutation accumulation on lifespan and aging rate, as well as a meta-analysis of morphometric, life history and metabolic data in 14 animal species (Dobler et al., 2014), which found a trend toward larger cytoplasmic effects in females.

**TABLE 1:** Examples of studies supporting mother’s curse.

Taxon	Reference(s)	Trait(s)
Flowering plants	Lewis. (1941); Budar et al. (2003); Yee et al. (2013), Case et al. (2016)	Fertility
*Drosophila*	Xu et al. (2008); Yee et al. (2013); Patel et al. (2016)	Fertility
	Camus and Dowling (2018)	Reproductive success
	Innocenti et al. (2011)	Gene expression
	Nagarajan-Radha et al. (2020)	Metabolic rate
	Sackton et al. (2003)	Mitochondrial enzyme activity
	Wolff et al. (2016)	Mitochondrial quantity
	Carnegie et al. (2021)	Morphology
	Aw et al. (2017)	Fertility, lifespan, lipid content, mitochondrial function
	Camus et al. (2015)	Fertility, aging
	Christie et al. (2004)	Lifespan
	Camus et al. (2012)	Lifespan, aging
Seed beetles	Dowling et al. (2007)	Fertility
	Immonen et al. (2016)	Reproductive aging
Copepods	Li et al. (2023)	Gene expression
Birds	Froman and Kirby (2005)	Fertility
Hares	Smith et al. (2010)	Fertility
Mice	Nakada et al. (2006)	Fertility
Humans	Ruiz-Pesini et al. (2000); Moore and Reijo-Pera. (2000); Holyoake et al. (2001)	Fertility
	Wallace et al. (1988); Milot et al. (2017)	Leber’s hereditary optical neuropathy, lifespan
	Li et al. (2015)	Lifespan

**TABLE 2:** Examples of studies not supporting mother’s curse.

Taxon	Reference(s)	Trait(s)
*Drosophila*	Hoekstra et al. (2018); Montooth et al. (2019)	Fertility
	Friberg and Dowling (2008)	Fertility
	Mossman et al. (2016a)	Development time
	Mossman et al. (2016b)	Gene expression
	Kurbalija Novičić et al. (2015)	Metabolic rate
	Nagarajan-Radha et al. (2019)	Lifespan
Seed beetles	Immonen et al. (2020)	Reproductive success
Copepods	Watson et al. (2022)	Fertility, lifespan
Mammals	Cayuela et al. (2023)	Lifespan, aging
Animals	Dobler et al. (2014)	Morphology, life history, metabolism

### Traits affected

Much support for the MC hypothesis (Table 1) comes from studies of fertility, which is expected to be particularly vulnerable to sex-biased selection (Hollocher and Wu, 1996; Turelli and Orr, 2000). However a subset of these studies measured only male fertility (Moore and Reijo-Pera, 2000; Ruiz-Pesini et al., 2000; Holyoake et al., 2001; Froman and Kirby, 2005; Nakada et al., 2006; Dowling et al., 2007; Yee et al., 2013), providing a weaker test of the hypothesis. Particularly striking results have been found for gene expression (Innocenti et al., 2011), with mitochondrial effects on nuclear gene expression being dramatically higher in males. It should be noted, however, that sex-specific gene expression may not result in sex differences in protein, much less in fitness, which is the true focus of the hypothesis. Other support comes from fitness-related traits that are particularly tied to energy demand, including metabolic rate (Nagarajan-Radha et al., 2020); mitochondrial traits (Sackton et al., 2003; Wolff et al., 2016; Aw et al., 2017) and LHON (Wallace et al., 1988; Milot et al., 2017). In one case, mitochondrial genetic variance was found in younger but not older animals (Wolff et al., 2016), in contrast to predictions of the mitochondrial theory of aging. However, numerous studies found support for MC on age-related traits, including studies of lifespan (Christie et al., 2004; Camus et al., 2012; Li et al., 2015; Aw et al., 2017; Milot et al., 2017), aging rate (Camus et al., 2012; Camus et al., 2015), and reproductive senescence (Immonen et al., 2016).

### Mixed results

Despite the variety of support for Mother’s Curse (Table 1), other studies on most of the same taxa and traits fail to find support (Table 2). This includes studies that find no sex differences in mitochondrial effects (Mossman et al., 2016a), sex-specific mitochondrial effects with no overall male bias (Kurbalija Novičić et al., 2015), and mitochondrial effects that are greater in *females* (Hoekstra et al., 2018; Montooth et al., 2019; Nagarajan-Radha et al., 2019; Watson et al., 2022). Many factors may contribute to this paucity of evidence, including factors that limit male mitochondrial load to begin with, as well as those that mask its expression.

## Why is there so little evidence for Mother’s Curse?

### Nuclear compensation makes male-harming mitochondrial mutations cryptic

When male-harming mitochondrial mutations slip through the sex-specific selective sieve, this should result in strong selection for compensatory nuclear mutations (Rand et al., 2004; Beekman et al., 2014). Such nuclear restorer mutations are well established in hermaphroditic plants (Budar et al., 2003), and also apparent in *Drosophila* (Clancy, 2008; Innocenti et al., 2011). While this nuclear compensation masks Mother’s Curse mutations, they may be unveiled in crosses that place them on a novel nuclear background. This may explain why much of the evidence for MC (Table 1) involves taxa amenable to experimental crosses, including *Drosophila*, the seed beetle *Callosobruchus maculatus*, and the copepod *T. californicus*. However, there is much debate about how often the evolutionary rate of nuclear compensation can keep pace with the rapid spread of MC alleles (Connallon et al., 2018; Dapper et al., 2023). Compensatory nuclear mutations can be expected to accumulate more rapidly on the Y chromosome, due to strict paternal transmission. This could negate the Mother’s Curse or even, in theory, create an opposing “Father’s Curse” fueled by Y-linked mutations that are beneficial to males but deleterious to females (Ågren et al., 2019).

### Accumulation of male-harming mutations counteracted by paternal leakage, inbreeding and kin selection

Paternal mitochondrial leakage is one factor that may slow the accumulation of Mother’s Curse mutations. While strict maternal cytoplasmic inheritance was thought to the norm in most eukaryotes, there are an increasing number of reports of detectable paternal leakage (Breton and Stewart, 2015; Kuijper et al., 2015). The proportion of offspring inheriting paternal alleles can vary from as low as 10^−6^ in mice to as high as 6% in *Silene vulgaris* and *Drosophila melanogaster* (reviewed in Dokianakis and Ladoukakis, 2014). Paternal leakage may be particularly common in interspecific crosses, where mechanisms maintaining strict maternal inheritance may break down (Dokianakis and Ladoukakis, 2014; Breton and Stewart, 2015). While the empirical effects of paternal mitochondrial leakage are unknown, modelling work suggests this leakage can dampen MC effects, particularly for mitochondrial mutations that are strongly beneficial for males and weakly deleterious for females (Kuijper et al., 2015). Low levels of paternal transmission do not, however, appear to eliminate MC effects, since several taxa showing support for the hypothesis (Table 1), have also been shown to exhibit paternal leakage (*D. melanogaster,* Nunes et al., 2013; *T. californicus;* Lee and Willett, 2022; mice; Gyllensten et al., 1991 and humans; Luo et al., 2018).

The spread of male-harming mutations can also be countered by both inbreeding and kin selection. Population genetic models show that even modest levels of inbreeding allow mitochondria to respond to selection on males, due to the correlation between male fertility and female fitness (Unckless and Herren, 2009; Wade and Brandvain, 2009). Note that technically this is positive assortative mating, rather than inbreeding, because there is no inbreeding for haploid mitochondrial genes (Hedrick, 2012). Selection on male mitochondrial fitness effects can also be expected to occur through kin selection, when males have indirect effects on the fitness of their sisters (Wade and Brandvain, 2009). Interestingly, both mechanisms can be predicted to increase the frequency of mitochondria harmful to *females* in some situations (Wade and Brandvain, 2009). Empirical work on *D. melanogaster* corroborates the complexities of selection on male mitochondria, with kin selection either increasing or decreasing MC effects depending on whether male-female relationships are competitive or cooperative (Keaney et al., 2020).

### Aberrant effects in interspecific studies

A possible reason for the mixed results may be unexpected effects of mitonuclear epistasis in interspecific hybrids. Indeed, only one of the studies supporting MC (Table 1) was based on interspecific hybrids (Sackton et al., 2003), while several studies failing to support the hypothesis (Table 2) were based on mitonuclear *Drosophila* genotypes in which the nuclear genomes are homozygous for one species, while the mtDNAs are from both intraspecific and interspecific origins (Mossman et al., 2016a; Mossman et al., 2016b; Hoekstra et al., 2018; Montooth et al., 2019). It is possible that placing a mtDNA haplotype on the extreme environment of a different species’ nuclear genome may expose unexpected, maladaptive mitochondrial effects (Dowling and Adrian, 2019; Dowling and Wolff, 2023). However, work on these same mitonuclear genotypes contradicts this prediction by showing that mtDNA mutations have greater effects within species than between species, suggesting that mitonuclear effects on fitness in this system result from segregating variation rather than fixed differences between species (Montooth et al., 2010; Rand and Mossman, 2020). Results contradicting MC predictions were also found in the copepod *T. californicus* (Watson et al., 2022), where some of the crosses were between populations that are more genetically divergent than hybridizing *Drosophila* species. Here, mitonuclear hybrids between the two most divergent populations did indeed show greater departures from MC predictions, with mitonuclear mismatch causing greater fitness problems in *females*.

### Context-dependent effects

Another factor limiting evidence for Mother’s Curse is that these effects seem to be highly context-dependent. In some cases, phenotypic effects of mtDNA can differ substantially across different nuclear backgrounds (Montooth et al., 2010; Meiklejohn et al., 2013; Patel et al., 2016; Rand and Mossman, 2020). For example, male sterility caused by a mtDNA mutation in *D. melanogaster* can be fully rescued on some nuclear backgrounds within the same species (Patel et al., 2016). Sex-specific mitochondrial effects can also be highly dependent on environment. Selection on sex differences is generally expected to be reduced under environmental stress (Berger et al., 2014; Connallon, 2015), and thermal stress has been specifically implicated in reducing the evolution of male-specific mitochondrial load (Immonen et al., 2020). Conversely, one study (Montooth et al., 2019) found male-harming mitonuclear effects to be expressed only under thermal stress. Further, this male harm could be rescued by diet (Montooth et al., 2019), indicating that sex-specific mitochondrial effects are both temperature and energy dependent. Such environmental sensitivity may vary between traits, with whole organism phenotypes such as fitness being proposed as more sensitive than gene expression (Rand and Mossman, 2020). This may contribute to discrepancies in sex-specific mitochondrial effects for different traits in the same taxa, such as those found in *Drosophila* (Mossman et al., 2016a; Mossman et al., 2016b) and the copepod *T. californicus* (Watson et al., 2022; Li et al., 2023).

### Demographic effects

In addition to all the factors above, evidence for Mother’s Curse may be limited by demographic factors that make some taxa less prone to accumulating male-harming mitochondrial mutations. MC effects are expected to be reduced in subdivided populations due to the maintenance of nuclear compensation (Munasinghe et al., 2022) as well as higher inbreeding (Zhang et al., 2012), which may counteract the accumulation of male-harming mutations. MC effects are also expected to be reduced in species with small effective population size, which are less able to maintain high levels of mtDNA-based fitness variance and also more prone to inbreeding (Unckless and Herren, 2009; Wade and Brandvain, 2009; Smith and Connallon, 2017). MC may therefore be less common in many vertebrates than in invertebrates such as *Drosophila* with high effective population sizes (Smith and Connallon, 2017).

## Conclusion

In sum, Mother’s Curse effects on lifespan and aging might be expected to be pervasive across eukaryotes, given the prevalence of maternal mitochondrial transmission and the established role of mitochondria in aging. And yet, evidence for such effects remains quite limited, especially in natural populations. This shortage of evidence can be attributed to a combination of factors, including compensatory nuclear mutations, mating systems, context dependency and demographic effects. Given the uneven evidence, particularly in animals, caution should be taken in expecting male harming mitochondria in a medical context. While the Mother’s Curse hypothesis provides testable predictions for understanding sex-specific mechanisms in experimental crosses, it appears to be of limited value in explaining actual sex differences in lifespan and aging.
